# Patterns of Skills Acquisition in Anesthesiologists During Simulated Interscalene Block Training on a Soft Embalmed Thiel Cadaver: Cohort Study

**DOI:** 10.2196/32840

**Published:** 2022-08-11

**Authors:** Graeme McLeod, Mel McKendrick, Tedis Tafili, Mateo Obregon, Ruth Neary, Ayman Mustafa, Pavan Raju, Donna Kean, Gary McKendrick, Tuesday McKendrick

**Affiliations:** 1 Ninewells Hospital Dundee United Kingdom; 2 University of Dundee Dundee United Kingdom; 3 Optomize Ltd Glasgow United Kingdom; 4 Heriot-Watt University Edinburgh United Kingdom; 5 Raigmore Hospital Inverness United Kingdom; 6 Barts and The London School of Medicine and Dentistry London United Kingdom

**Keywords:** regional anesthesia, ultrasonography, simulation, learning curves, eye tracking

## Abstract

**Background:**

The demand for regional anesthesia for major surgery has increased considerably, but only a small number of anesthesiologists can provide such care. Simulations may improve clinical performance. However, opportunities to rehearse procedures are limited, and the clinical educational outcomes prescribed by the Royal College of Anesthesiologists training curriculum 2021 are difficult to attain. Educational paradigms, such as mastery learning and dedicated practice, are increasingly being used to teach technical skills to enhance skills acquisition. Moreover, high-fidelity, resilient cadaver simulators are now available: the soft embalmed Thiel cadaver shows physical characteristics and functional alignment similar to those of patients. Tissue elasticity allows tissues to expand and relax, fluid to drain away, and hundreds of repeated injections to be tolerated without causing damage. Learning curves and their intra- and interindividual dynamics have not hitherto been measured on the Thiel cadaver simulator using the mastery learning and dedicated practice educational paradigm coupled with validated, quantitative metrics, such as checklists, eye tracking metrics, and self-rating scores.

**Objective:**

Our primary objective was to measure the learning slopes of the scanning and needling phases of an interscalene block conducted repeatedly on a soft embalmed Thiel cadaver over a 3-hour period of training.

**Methods:**

A total of 30 anesthesiologists, with a wide range of experience, conducted up to 60 ultrasound-guided interscalene blocks over 3 hours on the left side of 2 soft embalmed Thiel cadavers. The duration of the scanning and needling phases was defined as the time taken to perform all the steps correctly. The primary outcome was the best-fit linear slope of the log-log transformed time to complete each phase. Our secondary objectives were to measure preprocedural psychometrics, describe deviations from the learning slope, correlate scanning and needling phase data, characterize skills according to clinical grade, measure learning curves using objective eye gaze tracking and subjective self-rating measures, and use cluster analysis to categorize performance irrespective of grade.

**Results:**

The median (IQR; range) log-log learning slopes were −0.47 (−0.62 to −0.32; −0.96 to 0.30) and −0.23 (−0.34 to −0.19; −0.71 to 0.27) during the scanning and needling phases, respectively. Locally Weighted Scatterplot Smoother curves showed wide variability in within-participant performance. The learning slopes of the scanning and needling phases correlated: ρ=0.55 (0.23-0.76), *P*<.001, and ρ=−0.72 (−0.46 to −0.87), *P*<.001, respectively. Eye gaze fixation count and glance count during the scanning and needling phases best reflected block duration. Using clustering techniques, fixation count and glance were used to identify 4 distinct patterns of learning behavior.

**Conclusions:**

We quantified learning slopes by log-log transformation of the time taken to complete the scanning and needling phases of interscalene blocks and identified intraindividual and interindividual patterns of variability.

## Introduction

### Background

Ultrasound-guided regional anesthesia (UGRA) is a complex ultrasound-based needle intervention that requires extensive training to deliver safe, high-quality pain relief and the best possible perioperative outcomes [1]. The demand for UGRA has increased considerably during the COVID-19 pandemic because surgery can be conducted awake on insensate limbs, thus avoiding opioids, intubation, and ventilation [2]. However, there is a variation in the ability to perform UGRA among anesthesiologists. Training is sporadic: skills are first learned (and errors made) on patients, then honed intermittently over many years.

However, only a weak relationship exists between experience and actual measured performance [3] and potentially harmful behavior may be hidden in independent, isolated practice.

### Simulation Training

Simulation training may improve the UGRA performance [4]. Cadaver-based training courses are common but unstructured, and only basic skills are taught. Trainee:trainer ratios are high, and skills are acquired at different rates [5]. Thus, clinical educational outcomes prescribed by the Royal College of Anaesthetists training curriculum 2021 may be difficult to reach within short time frames [6,7].

A clear need exists for UGRA simulation training to compensate for the shortfall in clinical exposure to UGRA [8]; identify personal strengths and weaknesses using an expert performance approach [9]; gain insight into which personal characteristics and psychometric mechanisms impact performance; and categorize the learning patterns of a broad, general selection of anesthesiologists.

### Current Evidence

To date, our work has validated the physical and functional alignment of the Thiel embalmed cadaver simulator [10], developed and validated checklist and eye tracking metrics that reflect skills performance [11], introduced mastery learning and dedicated 1:1 training to regional anesthesia [5], measured learning curves using new needle technology [5], and showed translation of skills from cadavers to patients 3 weeks after training. We also used eye tracking to measure the performance of the simulators and patients [4]. Eye tracking measures the number of fixations (points at which the visual system takes in detailed information), duration (dwell time), and saccades (rapid movement between fixations). Our eye tracking data have shown construct validity and inverse correlation of fixations with the successful execution of checklist items [11].

The extended learning curves of anesthesiologists have not been established on a high-fidelity simulator of regional anesthesia using validated, quantitative metrics and educational paradigms associated with enhanced skill acquisition.

As the time taken to perform a task decreases with the number of repetitions of that task, learning follows the power law: log-log transformation yields a linear slope that can be easily interpreted.

### Primary Objective

Therefore, our primary objective was to measure the learning slopes of a wide range of anesthesiologists trained on an interscalene block on a soft embalmed Thiel cadaver, using an expert performance approach encompassing mastery learning and dedicated practice.

Thus, our primary outcome measure was the slope of the best-fit linear lines through log-log transformed data during the scanning and needling phases of the simulated interscalene nerve block.

## Methods

The study was conducted over a 3-week period at the Centre for Anatomy and Human Identification (CAHID) at the University of Dundee, governed by the Anatomy Act 2006 (Scotland). Reporting followed the Reporting Mastery Education Research in Medicine [12] guidelines, the Guidelines for Health Care Simulation Research [13], and the Strengthening the Reporting of Observational Studies in Epidemiology statements [14].

### Ethics Approval

The study was approved by the University of Dundee Non-Clinical Ethics Committee.

### Study Population

We invited anesthesiologists with a broad range of experience to participate in this study. They included trainees from years 1 to 7 in the East of Scotland School of Anesthesia, general consultant anesthesiologists, one expert regional anesthesiology fellow, who had completed the 7-year training program, and 2 consultant regional anesthesiologists who routinely practiced nerve blocks. We subdivided anesthesiology trainees into their 3 grades within the East of Scotland anesthesiology training program. Basic training occurred in years 1 to 2, intermediate training occurred in years 3 to 4, and higher training occurred in years 5 to 7.

All trainees and general consultants had basic or intermediate UGRA proficiency according to the Dreyfus and Dreyfus lexicons [15]. They had minimal background knowledge of regional anesthesia, struggled to address problems during nerve blocks using their own judgment, and were hesitant. They were representative of the population of anesthesiologists who may have infrequently conducted supervised or unsupervised nerve blocks and routinely used ultrasound to insert central venous lines. In contrast, consultant regional anesthesiologists practiced unsupervised nerve blocks on a regular basis, provided excellence with relative ease, recognized patterns, took responsibility for going beyond current standards, and developed ways of dealing with unique problems [15].

### Study Interventions

Before training, participants completed the self-reporting International Personality Item Pool (IPIP) and the State-Trait Anxiety Inventory tests. The IPIP consists of 50 statements describing 5 types of behavior: extraversion, agreeableness, conscientiousness, emotional stability, and intellect and imagination. Statements are answered using a 5-point categorical score from *very inaccurate* to *very accurate*. The State-Trait Anxiety Inventory consists of an S-Anxiety scale that uses 20 statements to evaluate on a 4-point descriptive scale how participants feel “right now, at this moment” and the T-Anxiety scale that uses 20 statements to assess on a 4-point descriptive scale how participants feel generally.

### Simulator

The Anatomy Scientific Officer selected 2 soft embalmed cadavers for this study. In CAHID, cadavers are soaked in vats for 6 months using the Thiel method with a mixture of salts and acids [16] and then stored for up to 3 years. Cadavers exhibit physical fidelity and functional alignment with simulated tasks [17]. Elasticity is similar to that of patients [18]: perineural injection distends and relaxes tissues, fluid drains away from the site, with minimal change in anatomy, allowing hundreds of repeated injections <0.5 mL [17] without cadaver damage.

The study was conducted in a quiet, well-lit, ventilated room in a mortuary at CAHID. An ultrasound machine (Zonare) was positioned on the right side of the neck, and the volunteers sat on the left side of the cadaver adjacent to the trainer. Volunteers wore SMI ETG 2w wireless eye tracking glasses (SensoMotoric Instruments). Psychologists sat behind a table at the head of the cadaver with study laptop computers that received live streaming of data from eye tracking glasses. Near-infrared light was projected onto the eyes, and integrated high frame-rate cameras detected the frequency and duration of eye gaze fixations, the period during which attention is relatively stable and focused at a given location; saccades, the rapid motion of the eye from one fixation to another; dwell time, the total amount of attention to an area of interest; and glances, the number of shifts in attention between the monitor and tools.

Before beginning each interscalene block, an eye tracking software calibration procedure was performed. Eye tracking data were masked from the viewpoints of the trainer and operator and downloaded to the raw data files.

### Study Procedure

Before the study started, trainers demonstrated the essential steps that were conducive to good practice and the errors that should be avoided. The participants started the study when they felt confident in doing so. The essential steps included preprocedural transducer handling and scanning skills; identification of target nerves; alignment of the needle to the transducer; visualization of the needle tip on needle movement and appropriate adjustment of its position when misaligned; observation of the needle tip during local anesthetic injection; recognition of tissue type and local anesthetic spread; and accidental intraneural injection.

Each volunteer conducted a maximum of 60 interscalene blocks within a 3-hour period. We chose this extended time frame to accommodate the wide range of competencies we expected to see and identify the dynamics of individual learning curves. Three experts performed 20 blocks because we expected them to perform at the top of their learning curve. We restricted the number of cadavers because we anticipated considerable variance in data both between and within participants over time as performance improved. Thus, the variance owing to the simulator was kept to the minimum possible. For the same reasons, we used only 2 expert regional anesthesiologists to supervise performance.

### Educational Approach

We applied the expert performance approach and used mastery learning and dedicated practices [6]. Participants had clear learning objectives and received continuous, proximate instructor feedback during each procedure [19]. All errors were identified by the trainer, communicated immediately, and steps outlined earlier were repeated by participants until successful, irrespective of time. Successful block was judged by the trainer as completion of all steps and accurate injection of a test dose of approximately 0.5-mL embalming solution between the C5 and C6 nerve roots. The injection times were recorded. As all tasks were completed successfully regardless of the time taken, scanning and needling durations were used as measures of block performance. All participants, including the experts, underwent the same training and testing. Thus, this was not a study comparing novices and experts, but a study designed to capture the range of performance of all participants. The demarcation between the scanning and needling phases was defined as the time of placement of the needle tip on the skin. A 5-minute break was taken every 30 minutes to minimize operator fatigue.

### Study Objectives

Our primary objective was to measure the learning slopes of the scanning and needling phases of the interscalene block conducted repeatedly on a soft embalmed Thiel cadaver over a 3-hour period of training.

Our secondary objectives were as follows:

- Measure preprocedural psychometrics
- Describe deviations from the learning slope
- Correlate scanning and needling phase data
- Measure learning curves using objective eye gaze tracking and subjective self-rating measures
- Use cluster analysis to categorize performance

### End Points

Our primary outcome measure was the slope of the best-fit linear lines using log-log transformed time data during the scanning and needling phases of the simulated interscalene nerve block.

Secondary end points were as follows:

- Eye metrics: eye gaze fixation count, relative amount of attention to the monitor (%), number of glances to the monitor, and relative amount of time (%) spent on the monitor (dwell) recorded during the scanning and needling phases.
- Self-confidence before and after each block [20] on a 10-point scale ranging from 1 “not at all confident” to 10 “extremely confident.”
- Anxiety was measured on a scale from 1 “extremely anxious” to 10 “extremely calm.”
- Global technical skills proficiency [21] after the first block was used as a baseline measure, then repeated after the final block. The assessment consisted of four scores: 1, unable to perform the procedure under supervision; 2, able to perform the procedure under supervision; 3, able to perform the procedure with minimum supervision (needed occasional help); and 4, competent to perform the procedure unsupervised (and could deal with any complications that arose).

Data were recorded during both the search and needle insertion phases of interscalene nerve block. The demarcation between the phases was defined as the time of needle tip placement on the skin. Eye tracking data were masked from the viewpoints of the trainer and operator and downloaded to the raw data files.

### Transformation of End Points

The ideal learning curve follows a power distribution. To analyze and interpret learning more easily, we log-transformed the data and plotted graphs. The primary and secondary endpoints were represented on the *y axis* and the procedure number on the *x axis.* The best-fit linear line was inserted through the data points. From each graph, we identified key features of the intercept (b), the slope (a), the SE of the slope, and the asymptote. The slope of the log-log plots constituted a measure of the rate of learning: a flat slope constituted no learning, and a steep slope indicated rapid learning.

The SE of each participant’s regression slope was taken as a measure of individual variability, and the asymptote, the average performance during the last 5 trials, was regarded as an indicator of the best performance.

### Statistical Analysis

Paired parametric data were analyzed using a paired 2-tailed *t* test and are presented as the difference between the means (95% CI). Paired nonparametric data were analyzed using the Wilcoxon test and are presented as the median of the differences (95% CI). The Kruskal-Wallis test was used to compare >2 groups. Linear models of log-log plots were assessed for fit using adjusted *R*^2^, the proportion of variation in the outcome explained by the predictor variables. The correlation between the intercept, slope, and asymptote in the scanning and needling phases was determined using the Spearman rank coefficient (ρ). Hierarchical clustering analysis was used to discriminate between the performances. The values were centered and scaled so that the magnitudes could be compared. Statistical analysis was performed using RStudio and GraphPad Prism.

### Power Analysis

As no previous anesthesia study had measured learning curves in such detail and the within-subject and between-subject errors of our data were not known, we made no prior assumptions about the data and recruited all willing participants.

## Results

### Participant Characteristics

In total, 33 anesthesiologists opted-in to the study and provided written informed consent. Participants 6, 26, and 29 did not participate in the study, and therefore, data from 30 participants were analyzed. Their personal characteristics are listed in Table 1. The median (IQR; range) ultrasound experience and anesthetic experience were 4 (3-6; 1-12) years and 4 (3-6; 1-29) years, respectively. The participants performed 51 (40-59; 28-60) blocks. IPIP scores were as follows: extraversion 28 (26-36; 11-43), agreeableness 38 (34-42; 23-48), conscientiousness 38 (34-42; 23-48), emotional stability 36 (29-40; 19-44), and intellect and imagination 35 (32-39; 23-45). The median (IQR; range) state anxiety score was 33 (27-37; 20-63) and trait anxiety score was 36 (22-42; 26-60).

**Table 1:** Participant characteristics.

Participant number	Age (years)	Sex	Grade	Anesthesia (year)	Repeat procedures (n)
1	33	Male	ST^a^ 6	6	58
2	34	Male	ST 5	6	49
3	28	Female	CT^b^ 2	2	28
4	27	Female	CT 1	1	30
5	32	Male	ST 4	4	51
7	27	Female	CT 1	1	50
8	29	Male	ST 3	3	40
9	30	Female	ST 4	4	34
10	35	Male	CT 2	2	30
11	30	Female	ST 4	4	37
12	29	Male	ST 4	4	44
13	31	Female	ST 6	6	58
14	30	Female	ST 4	4	60
15	30	Female	ST 3	3	54
16	30	Female	CT 2	2	28
17	30	Female	ST 3	3	60
18	29	Male	ST 4	4	60
19	32	Female	ST 4	4	47
20	26	Male	ST 4	4	53
21	32	Male	ST 4	4	60
22	36	Female	ST 4	4	60
23	35	Female	Con^c^	11	50
24	32	Male	ST 6	6	60
25	45	Male	Con	17	59
27	37	Female	Con	13	60
28	34	Female	ST 4	4	53
30	31	Male	CT 1	1	50
31	34	Female	Con	9	20
32	38	Male	Con	11	20
33	55	Male	Con	27	20

^a^ST: specialist trainee.

^b^CT: core medical trainee.

^c^Con: consultant.

### Cadaver Durability

Blocks were placed on the left neck of the 2 cadavers. No needle tracks were visualized on ultrasound images with repeated injections. We reported the durability of the first cadaver in this study in a previous publication [17]. It tolerated 934 interscalene blocks over 10 days without any discernible accumulation of perineural fluid compared with right-sided ultrasound control images. Tissue integrity had been attributed to tissue elasticity similar to that measured in humans [17,18].

### Learning Slopes

**Figure 1:**
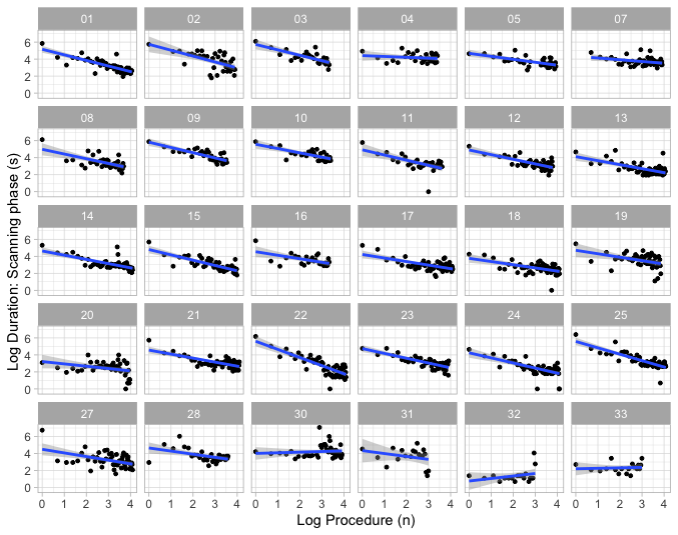
Best-fit linear learning slopes demonstrated on log-log transformed (power) model from participants 1 to 33 during search phase of simulated interscalene block. Participants 6, 26, and 29 are excluded. Log time (duration) taken to complete all steps on y-axis, and log sequence of blocks (1 to 4) the x-axis. The blue straight line is the best-fit line through the data. The 95% CIs about the slope are shown in light gray.

We plotted the log times taken for 30 participants to complete the scanning and needling phases and the log number of repetitions over 3 hours. Figure 1 shows the best-fit linear learning slopes for scanning time. Performance is indicated by the linear slope (95% CI), intercept (95% CI), error, and asymptote of the best-fit line passing through log-log converted data.

During the scanning phase, the median (IQR; range) slope was −0.47 (−0.62 to −0.32; −0.96 to 0.30) and median (IQR; range) log intercept was 4.70 (4.30-5.00; 0.76-5.80). During the needling phase, median (IQR; range) slope was −0.23 (−0.34 to −0.19; −0.71 to 0.27) and the median (IQR; range) log intercept was 4.20 (3.90-4.50; 2.90-5.80). Both the slope and SE of expert anesthesiologists (participant numbers 31, 32, and 33) notably had a relatively flat slope with little variation during scanning (Figure 1) and needling. Two novice anesthesiologists (participant numbers 12 and 17) also had a flat slope, but this was associated with marked variability, indicative of poor performance. The results are shown in Table 2.

**Table 2:** Individual learning slope data for scanning and needling time.

Patient number	Scanning phase						Needling phase				
	Line intercept	Slope (SE; 95% CI)	Log asymptote	Adjusted *R*^2^	LOESS^a^	Line intercept		Slope (SE; 95% CI)	Log asymptote	Adjusted *R*^2^	LOESS
1	5.16 (4.75 to 5.56)	−0.66 (0.06; −0.78 to 0.53)	2.55	0.66	→^b^	4.44 (3.96 to 4.98)		−0.31 (0.07; −0.46 to 0.16)	3.48	0.22	→ ↓
2	5.74 (4.81 to 6.66)	−0.69 (0.14; −1.00 to 0.39)	3.19	0.35	→ ↓	4.16 (3.58 to 4.73)		−0.20 (0.09; 0.39 to 0.02)	3.37	0.07	→ ↓
3	5.73 (5.14 to 6.31)	−0.64 (0.12; −0.87 to 0.41)	3.53	0.54	→ ↑	5.26 (4.64 to 5.88)		−0.50 (0.12; 0.72 to 0.26)	4.03	0.39	→ ↑
4	4.41 (3.81 to 5.01)	−0.10 (0.11; −0.33 to 0.13)	4.18	0.01	→ ↑	4.49 (3.70 to 5.29)		−0.19 (0.15; 0.49 to 0.13)	3.71	0.02	→ ↑
5	4.67 (4.25 to 5.10)	−0.35 (0.07; −0.49 to 0.21)	3.31	0.40	→	2.85 (2.26 to 3.44)		0.18 (0.01; 0.01 to 0.37)	3.76	0.07	↑
7	4.33 (3.82 to 4.85)	−0.21 (0.08; −0.37 to −0.04)	3.66	0.10	→ ↑	4.54 (3.70 to 5.29)		−0.28 (0.11; 0.51 to 0.05)	4.03	0.10	→ ↑
8	4.99 (4.28 to 5.69)	−0.55 (0.12; −0.80 to 0.31)	2.92	0.37	→ ↑	5.25 (4.53 to 5.94)		−0.35 (0.12; 0.59 to 0.11)	4.10	0.19	→ ↑
9	5.82 (5.38 to 6.28)	−0.62 (0.08; −0.78 to −0.45)	3.89	0.64	→	4.43 (3.93 to 4.93)		−0.27 (0.09; 0.45 to 0.09)	3.69	0.20	→ ↓
10	5.58 (5.06 to 6.11)	−0.51 (0.10; −0.70 to −0.31)	3.85	0.49	→	4.20 (3.66 to 4.75)		−0.23 (0.10; 0.44 to 0.03)	3.31	0.14	→ ↓
11	4.92 (4.18 to 5.67)	−0.61 (0.13; −0.87 to −0.34)	3.14	0.37	→	4.24 (3.69 to 4.80)		−0.28 (0.10; 0.48 to 0.08)	3.14	0.17	→
12	4.91 (4.40 to 5.42)	−0.55 (0.08; −0.72 to −0.38)	3.34	0.50	→	4.29 (3.64 to 4.95)		−0.07 (0.10; 0.29 to 0.15)	4.05	0.42	→
13	4.12 (3.62 to 4.62)	−0.47 (0.08; −0.63 to −0.32)	2.42	0.39	→ ↑	4.56 (4.12 to 4.99)		−0.44 (0.07; 0.58 to 0.30)	2.89	0.42	→
14	4.63 (4.20 to 5.06)	0.50 (0.07; −0.63 to −0.36)	2.43	0.49	→	4.30 4.01 to 4.71)		−0.39 (0.05; 0.50 to 0.28)	2.81	0.47	→
15	4.80 (4.34 to 5.25)	−0.62 (0.07; −0.77 to −0.48)	2.72	0.61	→ ↑	4.61 (4.22 to 4.98)		−0.42 (0.06; 0.50 to 0.30)	3.18	0.51	→
16	4.54 (3.89 to 5.19)	−0.42 (0.12; −0.67 to 0.16)	3.27	0.27	→ ↑	4.11 3.55 to 4.67)		−0.20 (0.11; 0.42 to 0.02)	3.33	0.10	→ ↑
17	4.22 (3.78 to 4.68)	0.42 (0.07; −0.57 to −0.29)	2..61	0.39	→ ↑	4.16 (3.65 to 4.68)		−0.20 (0.08; 0.36 to 0.04)	3.16	0.08	→ ↑
18	3.40 (2.75 to 4.05)	−0.27 (0.10; −0.46 to 0.08)	2.38	0.13	→	3.64 (3.20 to 4.08)		−0.09 (0.06; 0.22 to 0.04)	3.23	0.02	→ ↑
19	4.71 (3.89 to 5.53)	−0.41 (0.13; −0.68 to 0.10)	3.30	0.18	→ ↑ →	4.72 (4.05 to 5.38)		−0.34 (0.11; 0.56 to 0.12)	3.59	0.12	→ ↑ →
20	3.22 (2.42 to 4.01)	−0.27 (0.13; −0.53 to 0.02)	1..34	0.09	→ ↓	3.56 (3.03 to 4.10)		−0.19 (0.08; 0.37 to 0.02)	1.92	0.09	→ ↓
21	4.57 (4.16 to 4.97)	−0.47 (0.06; −0.60 to 0.35)	2.89	0.50	→ ↑	4.45 (3.65 to 5.24)		−0.23 (0.12; 0.46 to 0.01)	3.60	0.05	→ ↑
22	5.61 (5.00 to 6.22)	−0.96 (0.09; −1.14 to −0.77)	2.17	0.64	→	5.76 (5.18 to 6.34)		−0.71 (0.07; 0.88 to 0.53)	2.83	0.52	→
23	4.74 (4.33 to 5.16)	−0.57 (0.07; −0.70 to −0.44)	2.92	0.60	→	4.20 (3.66 to 4.74)		−0.22 (0.09; 0.39 to 0.04)	3.89	0.09	→ ↑
24	4.76 (4.11 to 5.42)	−0.75 (0.10; −0.95 to 0.55)	1.76	0.48	→	3.93 (3.61 to 4.26)		−0.23 (0.05; 0.32 to 0.12)	3.24	0.27	→
25	5.58 (5.14 to 6.03)	−0.75 (0.07; −0.90 to −0.62)	2.92	0.68	→	4.14 (3.79 to 4.49)		−0.30 (0.05; 0.41 to 0.20)	2.76	0.35	→
27	4.49 (3.78 to 5.20)	−0.42 (0.11; −0.64 to 0.21)	2.47	0.20	→ ↓	3.37 (2.86 to 3.82)		0.27 (0.08; 0.42 to 0.11)	2.47	0.16	→
28	4.65 (4.00 to 5.31)	−0.37 (0.12; −0.60 to −0.13)	3.53	0.21	↑ ↓	4.38 (3.80 to 4.95)		−0.18 (0.09; 0.36 to 0.01)	4.15	0.07	→ ↑
30	4.01 (3.27 to 4.75)	0.07 (0.12; −0.16 to 0.31)	4.14	0.01	→ ↑	3.29 (2.51 to 4.06)		−0.09 (0.13; −0.35 to 0.16)	3.58	0.00	→
31	4.34 (2.96 to 5.72)	−0.34 (0.23; −0.95 to 0.27)	3.43	0.20	→ ↑	3.55 (3.26 to 3.88)		−0.40 (0.06; −0.54 to 0.27)	2.43	0.62	→ ↑
32	0.76 (−0.28 to 1.80)	0.30 (0.22; −0.17 to 0.76)	2.81	0.40	→ ↑	3.11 (2.64 to 3.57)		0.02 (0.10; −0.18 to 0.24)	3.06	0.05	→ ↑
33	2.20 (1.43 to 2.98)	0.05 (0.16; −0.29 to 0.40)	2.73	0.05	→ ↑	3.92 (3.56 to 4.28)		−0.19 (0.07; −0.35 to 0.04)	3.37	0.05	→

^a^LOESS: Locally Weighted Scatterplot Smoother.

^b^LOESS fit described with arrows: → indicates good approximate fit to slope, ↓ indicates LOESS line persistently below the slope and accelerated learning, and ↑ indicates LOESS line persistently above the slope and slowed learning. Combinations of ↑, ↓, and → give an overview of learning dynamics.

### Data Variability

**Figure 2:**
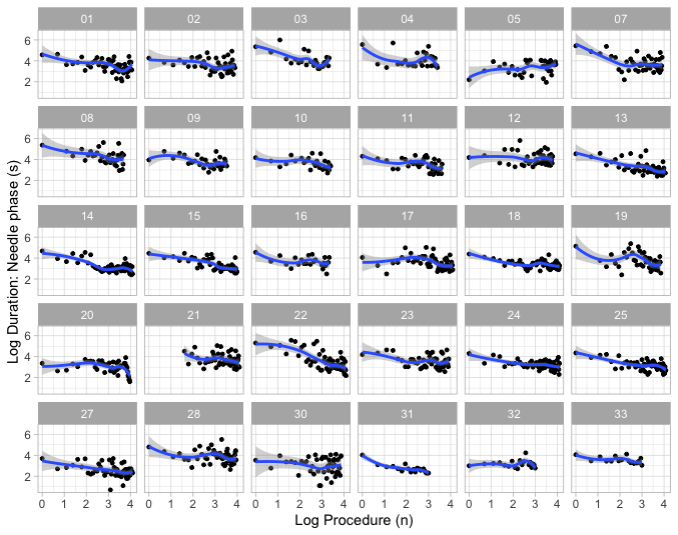
Best-fit Locally Weighted Scatterplot Smoother learning slopes demonstrated on log-log transformed (power) model from participants 1 to 33 during needling phase of simulated interscalene block. Participants 6, 26, and 29 were excluded. Log time (duration) taken to complete all steps on y-axis, and log sequence of blocks (1 to 4) the x-axis. The blue straight line is the best-fit line through the data. The 95% CIs about the slope are shown in light gray.

Locally Weighted Scatterplot Smoother (LOESS) best-fit lines illustrate the dynamics of learning during the search phase and during the needling phase (Figure 2) and are summarized in Table 2. In the scanning phase, the slope of learning remained close to a straight line in 12 participants, dropped below the line in 4 participants (indicating improved performance), rose above the line in 13 participants (indicating slowed learning), and moved in a complex manner above and below the learning slope in 1 participant. During the needling phase, improvements approximated the slope of learning in 12 participants, improved in 6, and worsened in 11. One participant exhibited a complex pattern. Of the participants, 60% (18/30) showed similar patterns in the scanning and needling phases.

In Table 2, columns show participant characteristics and linear and LOESS best-fits. Linear model characteristics include intercept on y axis, and slope and described using adjusted *R*^2^. LOESS fit is described using arrows: → indicates good approximate fit to slope, ↓ indicates LOESS line persistently below the slope and accelerated learning, and ↑ indicates LOESS line persistently above the slope and slowed learning. Combinations of ↑, ↓, and → give an overview of learning dynamics.

### Association Between Scanning and Needling Phases

The correlations between the intercept, slope, variation of the slope, and asymptote in the scanning and needling phases are shown in Table 3. The greater the initial time taken (intercept) to perform the interscalene block, the greater the rate of learning in the scanning and needling phases ρ=−0.87 (−0.94 to −0.73), *P*<.001, and ρ=−0.45 (−0.70 to −0.09), *P*=.01.

The learning slopes of the scanning and needling phases correlated; ρ=0.55 (0.23-0.76), *P*<.001; and ρ=−0.72 (−0.46 to −0.87), *P*<.001, respectively.

**Table 3:** Correlation (ρ) between markers of learning in scanning and needling phases. Markers include the learning slope, the best-fit linear line through log-log data; the variability of the slope represented by the SE; and the asymptote, the mean of the last 5 times taken to complete the procedure.

			Scanning									Needling				
			Line intercept		Slope		SE		Line asymptote		Line intercept			Slope		SE
**Scanning**																
	Slope (95% CI); *P* value	−0.87 (−0.94 to −0.73); <.001		N/A^a^		N/A		N/A		N/A			N/A		N/A	
	SE (95% CI); *P* value	−0.24 (−0.56 to 0.14); .20		0.38 (0.01 to 0.66); .04		N/A		N/A		N/A			N/A		N/A	
	Line asymptote (95% CI); *P* value	0.23 (0.15 to 0.56); .21		0.19 (0.18 to 0.53); .29		0.20 (−0.19 to −0.53); .30		N/A		N/A			N/A		N/A	
**Needling**																
	Line intercept (95% CI); *P* value	0.48 (0.14 to 0.72); .007		−0.44 (0.70 to −0.9); .01		−0.27 (0.58 to 0.11); .15		0.10 (−0.28 to 0.46); .59		N/A			N/A		N/A	
	Slope (95% CI); *P* value	−0.45 (−0.70 to −0.09); .01		0.55 (0.23 to 0.76); .001		0.17 (−0.21 to 0.51); .37		0.10 (−0.28 to 0.45); .61		−0.71 (−0.86 to −0.46); <.001			N/A		N/A	
	SE (95% CI); *P* value	0.01 (−0.36 to 0.38); .96		0.24 (0.14 to 0.56); .20		0.30 (−0.08 to 0.60); .11		0.57 (0.26 to 0.78); <.001		0.32 (−0.06 to 0.61); .09			0.10 (−0.28 to 0.45); .60		N/A	
	Line asymptote (95% CI); *P* value	0.26 (−0.12 to 0.57); .16		0.03 (0.34 to 0.39); .87		−0.12 (−0.47 to 0.26); .54		0.60 (0.29 to 0.79); .001		0.39 (−0.002 to 0.65); .04			0.54 (−0.24 to 0.49); .46		0.54 (0.21 to 0.76); .002	

^a^N/A: not applicable.

### Effect of Grade of Anesthesiologist on Scanning and Needling

**Figure 3:**
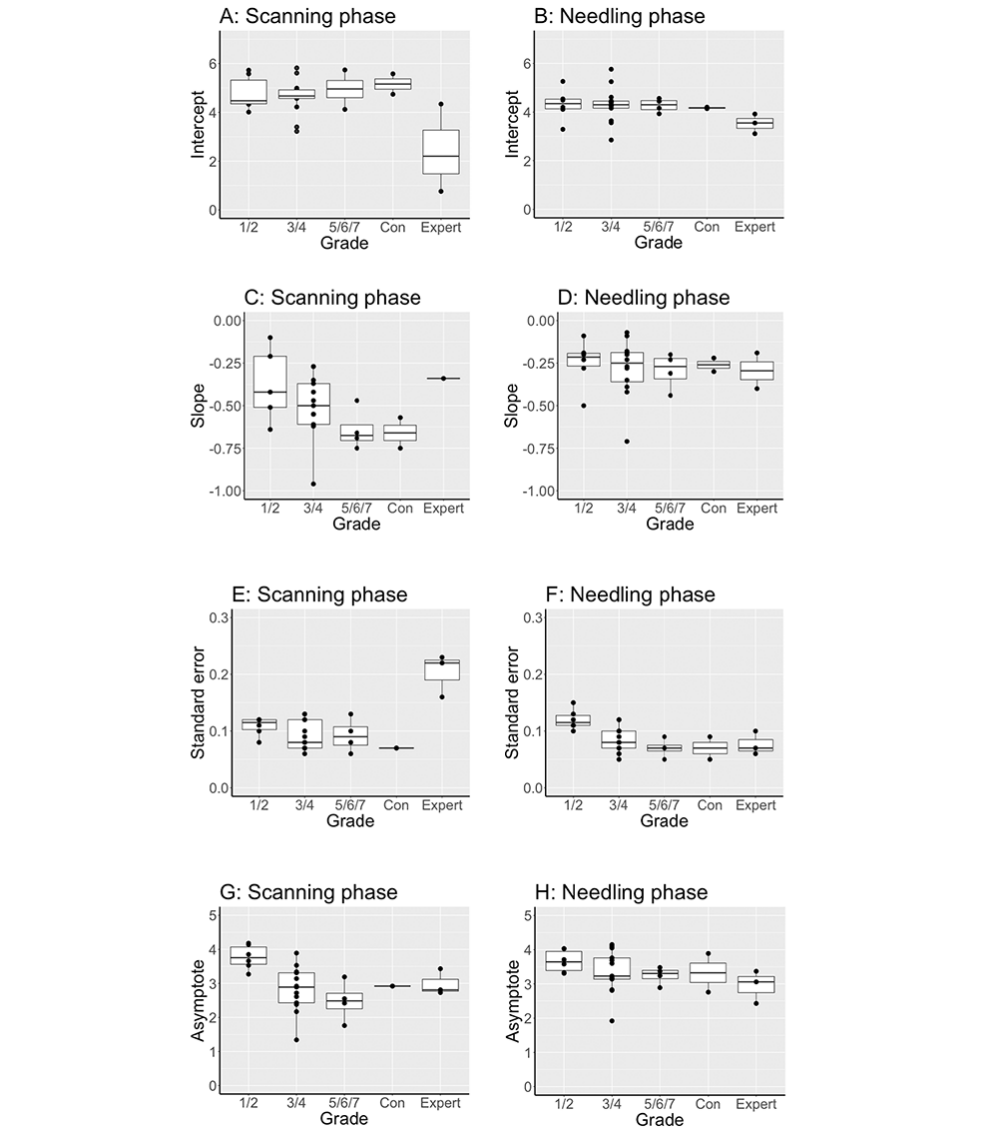
Grade. Experts had a flatter slope but greater variability during scanning, but less variability during needling (all comparisons *P*=.02). Novice anesthesiology trainees correspond to years 1 to 2 (1/2); intermediate anesthesiology trainees to years 3 to 4 (3/4); and higher anesthesiology trainees to years 5 to 7 (5/6/7). Consultant non-expert anesthesiologists designated as “Con”.

The relationship between anesthesiology grade and learning is shown in Figure 3.

The experts had a flatter slope but greater variability during scanning but less variability during needling (all comparisons *P*=.02).

### Secondary End Points

**Figure 4:**
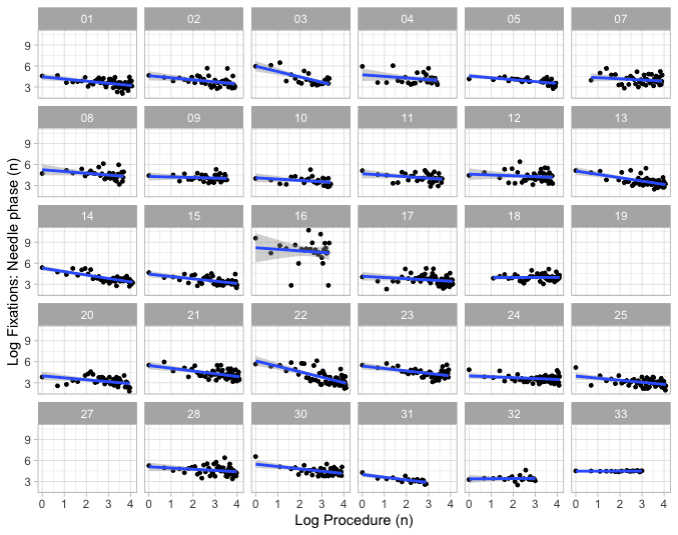
Eye gaze fixation count. Best-fit linear learning slopes demonstrated on log-log transformed (power) model from participants 1 to 33 during search phase of simulated interscalene block. Participants 6, 26, and 29 were excluded. Fixation count on y-axis, and log sequence of blocks (1 to 4) the x-axis. The blue straight line is the best-fit line through the data. The 95% CI about the slope are shown in light gray.

Linear slopes and LOESS best-fit lines were generated for our secondary endpoints (fixation count, relative fixation to the monitor [%], glance count and relative dwell time [%], self-confidence, and anxiety scores). An example of using the best-fit linear slopes of eye fixation counts in the search phase is shown in Figure 4.

### Data Distribution

**Figure 5:**
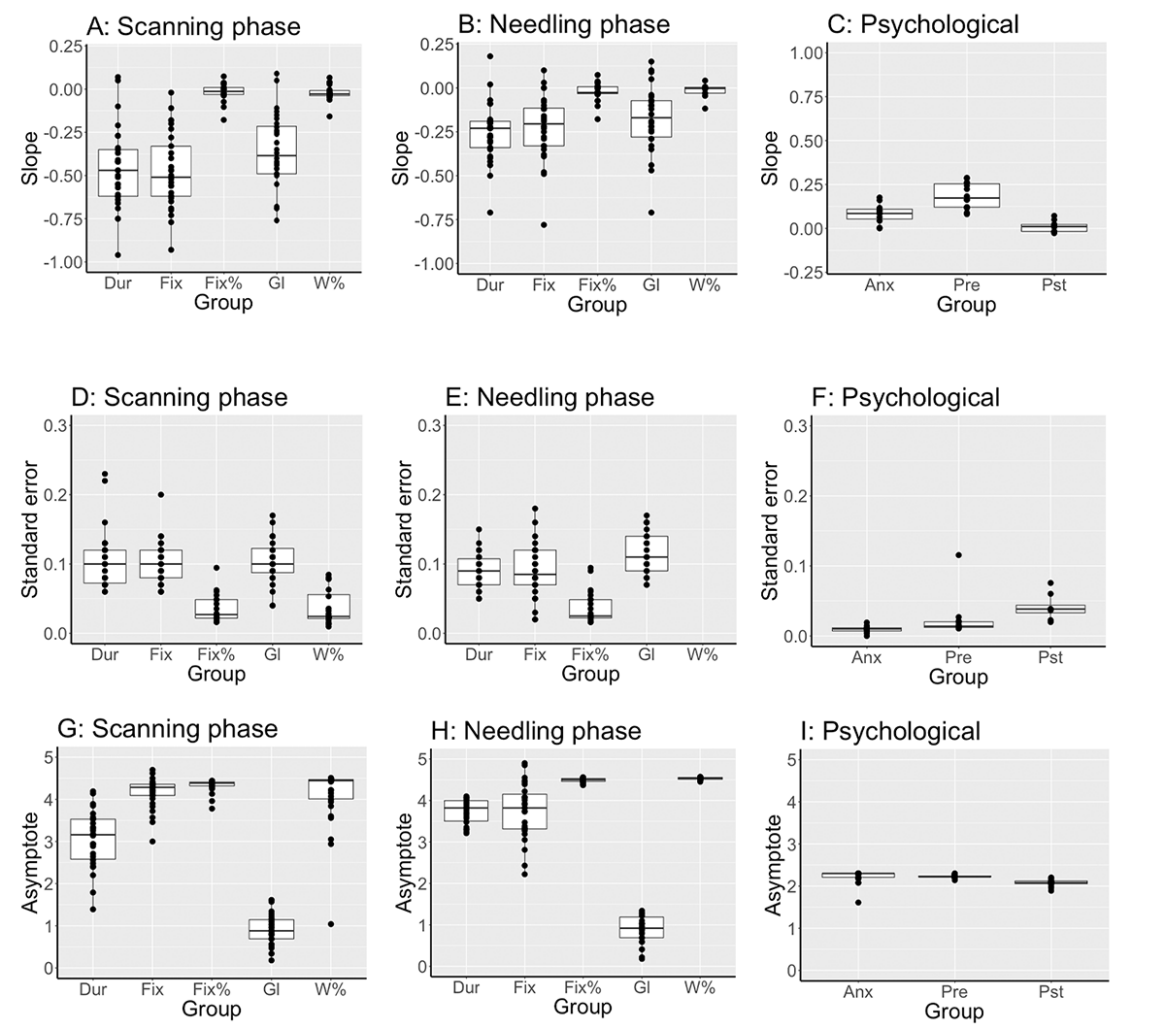
Slope estimate, slope standard error and asymptote of the primary end point, duration (Dur) and secondary end-points, median (IQR [range]). Secondary end-points include: eye gaze fixation count (Fix), relative fixation to the monitor (Fix%), glance count (G), and relative dwell time (W%) during the scanning and needling phases; and pre block anxiety (Anx) and self-confidence (Pre) and post block self-confidence (Pst). Large variation in effect with duration, fixation and glance count but not psychological variables.

The distribution of slope estimate, slope SE, and asymptote data (indicated by median (IQR; range) are shown for the primary endpoint (duration) and secondary endpoints (Figure 5). Eye gaze fixation count and glance count during the scanning and needling phases best reflected the median (IQR) block duration. In contrast, relative fixation on the monitor (%), relative dwell time (%), self-confidence, and anxiety scores showed little variation. The wide distribution of fixation count and glance count reflected the wide distribution of time to complete scanning and needling. Therefore, block duration, fixation count, and glance count were chosen as quantitative markers for performance discrimination.

### Correlation Between End Points

**Figure 6:**
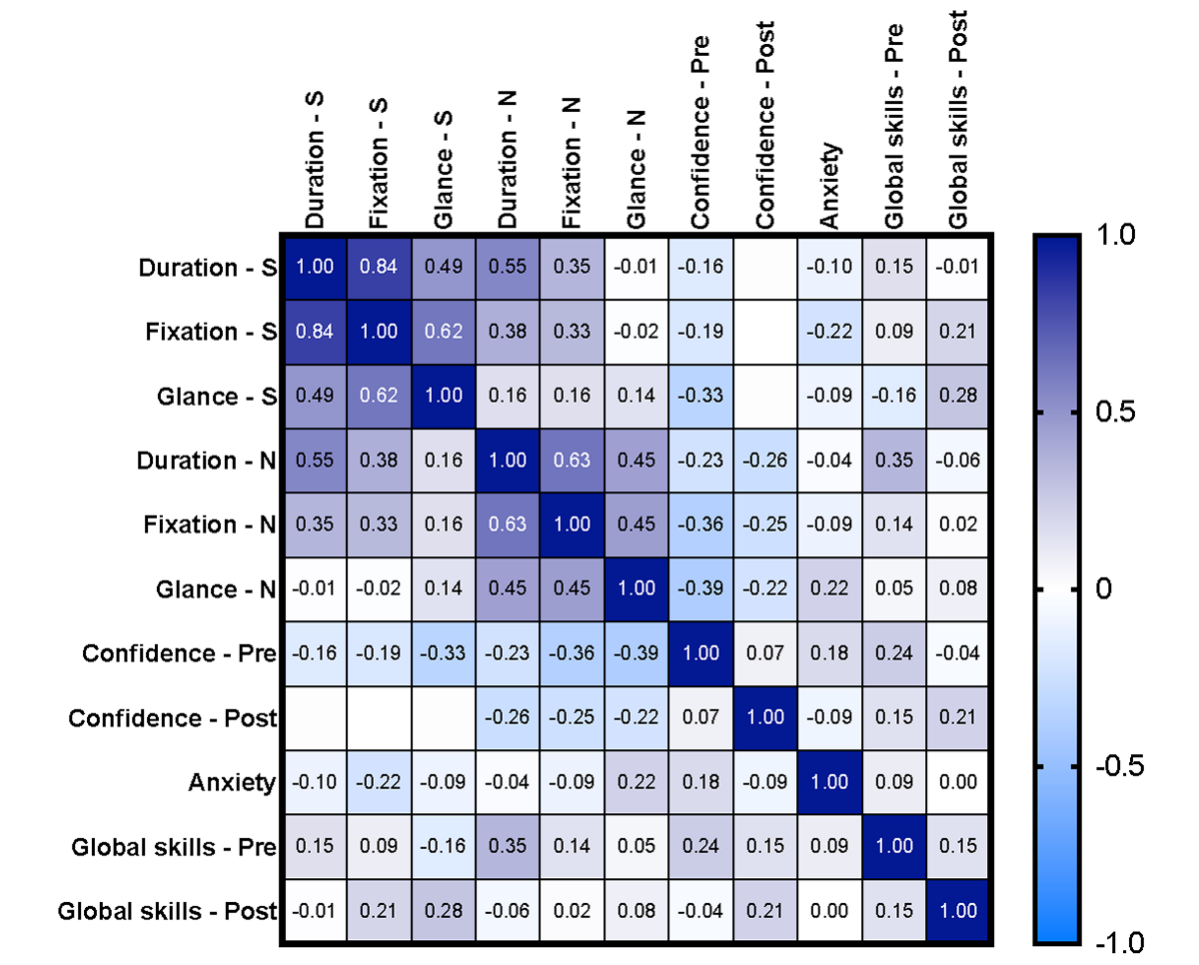
Correlation (ranging from −1 to +1) between procedural duration, fixation count, and glance count in the scanning (S) and needling (N) phases; mean pre- and postprocedural confidence; procedural anxiety; and initial and final proficiency. The scale indicated on the right is color mapped in shades of purple from 0 to +1 and shades of blue from 0 to −1. The largest correlations existed among procedural duration, fixation, and glance count in both the scanning and needling phases.

Learning slopes (duration) correlated with eye fixation and glance slopes in both the scanning and needling phases (Figure 6) but not with confidence, anxiety, or global skills scores.

### Clustering

**Figure 7:**
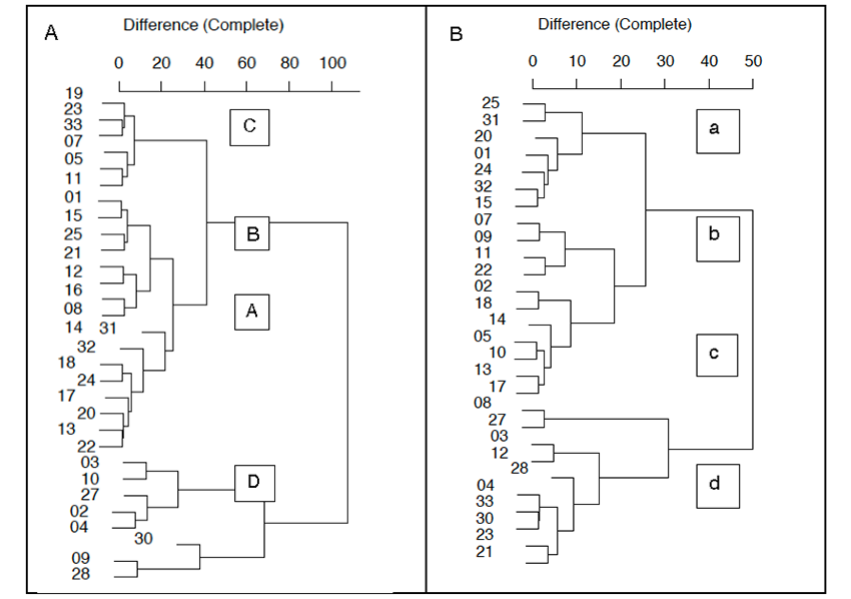
Dendrograms created by cluster analysis of preprocedural and procedural fixation and glance counts. Search phase (groups A, B, C, D) and needle phase (groups a, b, c, d).

A cluster analysis of preprocedural and procedural fixation and glance counts (Figure 7) identified 4 distinct performance groups within both the search and needling phases.

**Figure 8:**
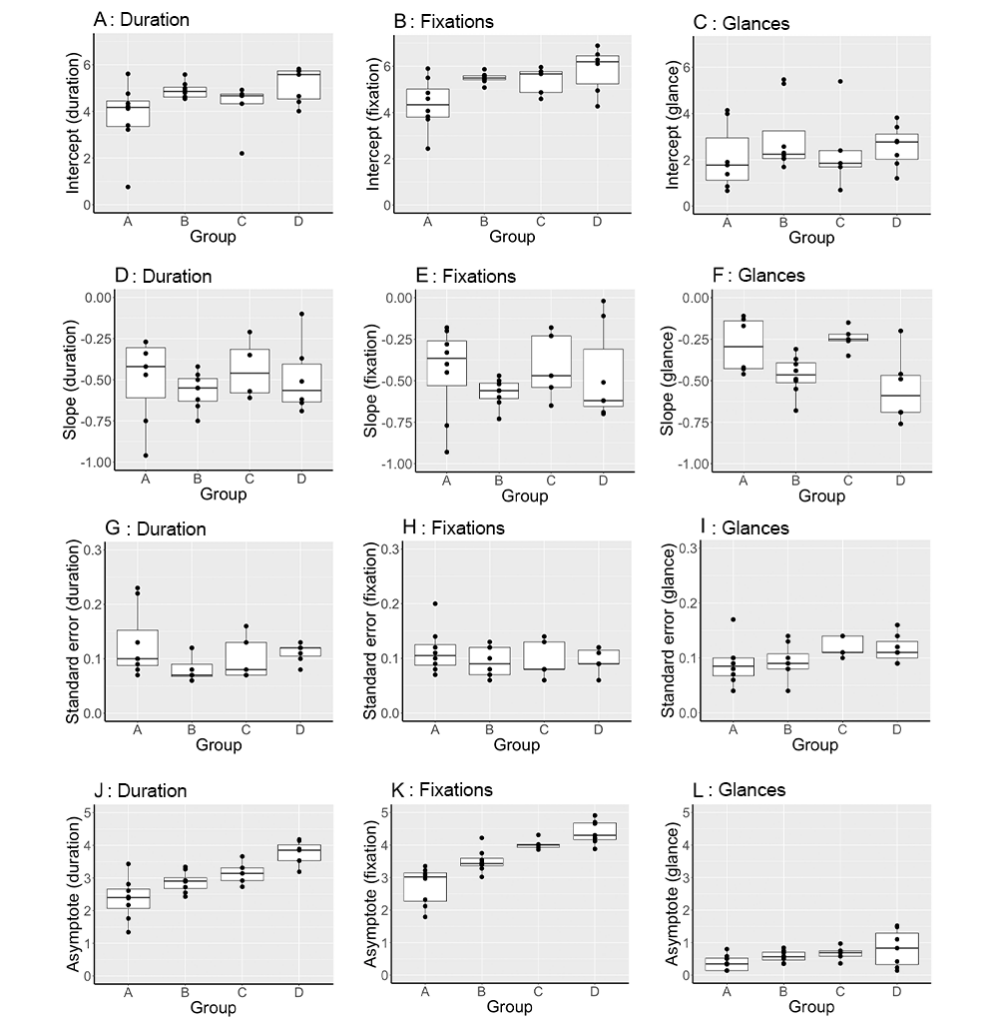
Characteristics of scanning phase learning slopes (procedure duration, eye fixation and glance) according to groups defined by cluster analysis. Characteristics include intercept, slope standard error and asymptote. Better performance was associated with reductions in: the asymptote of procedure duration (image J), (χ^2^ 17.0, *P*<.001); the intercept (image B), (χ^2^ 9.5, *P*=.02) and asymptote (image K), (χ^2^ 21.2, *P*<.001) of eye gaze fixations; and the learning slope of eye glances (image F), (χ^2^ 9.3, *P*=.03).

**Figure 9:**
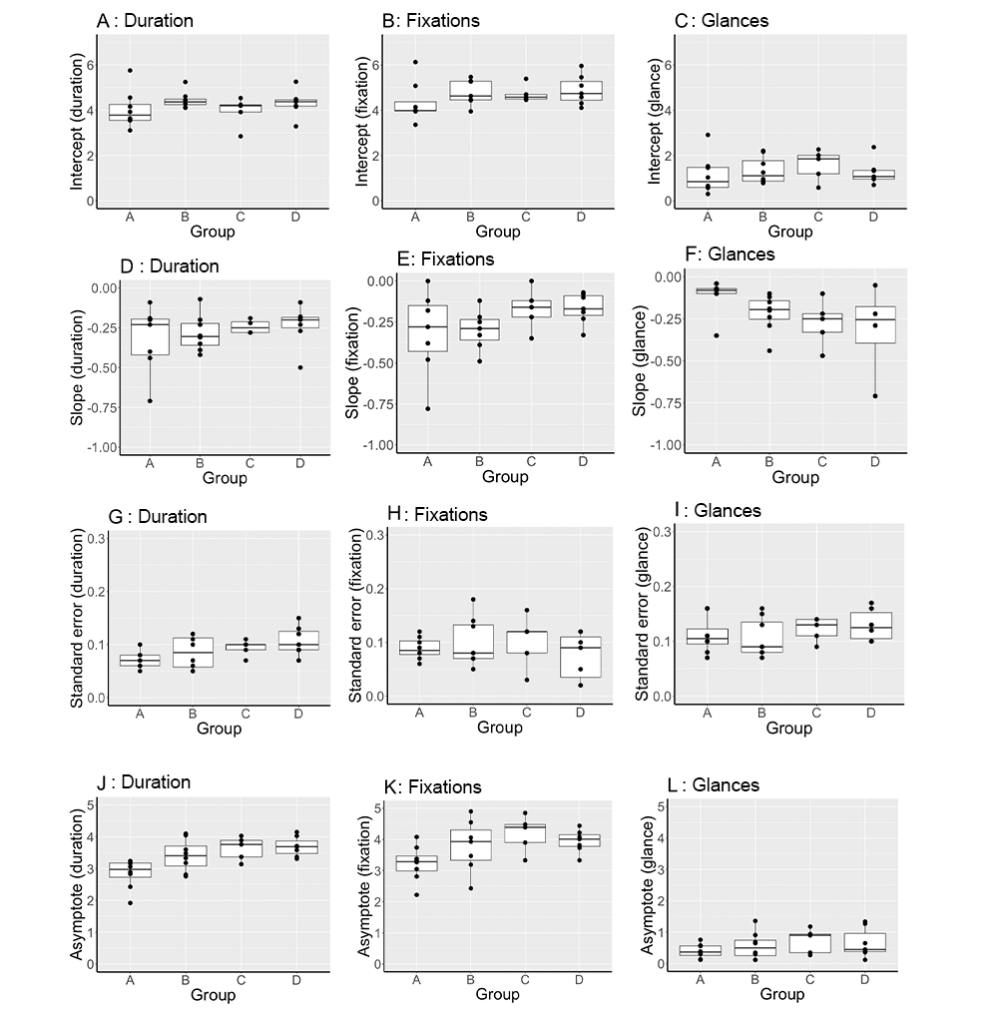
Characteristics (intercept, slope, error and asymptote) of the learning slopes for duration, eye fixations and eye glances during the needling phase, according to groups defined by cluster analysis. better performance was associated with reductions in: the standard error (image H) (χ^2^ 9.6, *P* .02); and asymptote of procedure duration (image K) (χ^2^ 14.4, *P*=.002); and the intercept (image B) (χ^2^ 12.8, *P*=.005) and asymptote of eye gaze fixations (image L) (χ^2^ 7.9, *P*=.04).

Groups were ranked according to performance (from best to worst) as A, B, C, and D in the scanning phase (Figure 8) and a, b, c, and d in the needling phase (Figure 9). Figure 9 outlines the characteristics (intercept, slope, error, and asymptote) of the learning slopes for the duration, eye fixations, and eye glances during the scanning phase. Distinct performance trends are observed for the best to worst performance. For example, better performance was associated with reductions in the asymptote of procedure duration (image J; χ^2^_3_=17.0; *P*<.001); the intercept (image B; χ^2^_3_=9.5; *P*=.02) and asymptote (image K; χ^2^_3_=21.2; *P*<.001) of eye gaze fixations; and the learning slope of eye glances (image F; χ^2^_3_=9.3; *P*=.03).

Figure 9 outlines the characteristics (intercept, slope, error, and asymptote) of the learning slopes for duration, eye fixations, and eye glances during the needling phase according to groups defined by cluster analysis. The same trends in performance for the best to worst performance were observed using the intercepts and asymptotes as in the scanning phase. For example, better performance was associated with reductions in the SE (image H; χ^2^_3_=9.6; *P*=.02), asymptote of procedure duration (image K; χ^2^_3_=14.4; *P*=.002), intercept (image B; χ^2^_3_=12.8; *P*=.005), and asymptote of eye gaze fixations (image L; χ^2^_3_=7.9; *P*=.04).

## Discussion

### Principal Findings

We characterized the individual learning slopes of 30 anesthesiologists performing simulated interscalene blocks using log-log transformations of procedure-time data. The dynamic nature of learning was captured by LOESS best-fit lines. Needling performance paralleled scanning performance. Expert anesthesiologists were characterized by consistent and stable performance, whereas novice anesthesiologists took longer, and their performance varied. We identified 4 disparate skill groups for scanning and needling. The discrimination of performance using eye gaze fixation count and glance count reflected the discrimination of performance using procedural time.

### Strengths and Weaknesses

The strengths of our study were its educational and statistical approaches, application of quantitative metrics, and the use of a validated high-fidelity simulator.

### Educational Approach

First, we applied mastery learning and dedicated practice as part of the expert performance approach to each preprocedural and procedural step, rather than the nerve block as a whole. Mastery learning is increasingly used in medical schools for skills training [22], and a recent review has recommended the introduction of deliberate practice into anesthesia teaching [23]. Unlike traditional assessment approaches that set a threshold for pass or fail over a set time, mastery learning endeavors to achieve a predefined skill level for all participants, irrespective of time [24]. Thus, in our study, all blocks were conducted successfully, irrespective of training duration. In this way, the measurement of duration was regarded as a marker of block quality because all items were completed. However, a weakness of our approach is that we failed to measure the number of errors made. Only one study that investigated UGRA training measured tasks and errors [25]. In future work, we intend to test trainees using video analysis of errors, including those that may cause harm to the patient.

### Statistical Approach

Second, we demonstrated that skill acquisition follows the power law of learning [26]. Although log-log conversion enabled a linear fit through all data sets, intraparticipant variability still occurred over time. Individual dynamics were most apparent using LOESS curves in both the scanning and needling phases and revealed a number of different learning patterns. Owing to the complexity of these patterns, we intend to analyze data sets in the future using nonlinear mixed-effects models, Bayesian methods, and machine learning [27,28]. Advanced modeling will enable us to better fit individual learning curves and capture both within- and between-participant variability in initial performance and any deterioration in performance due to fatigue.

Cluster analysis enabled us to discriminate performance irrespective of the grade (specialist trainee [ST]) and year of training. We identified 6 participants (participant numbers 13 [ST6], 17 [ST3], 18 [ST4], 20 [ST4], 22 [ST4], and 24 [ST6]) who matched expert scanning performance and 5 participants (participant numbers 1 [ST6], 15 [ST3], 20 [ST4], 24 [ST6], and 25 [consultants]) who matched expert needling performance. These best learners consistently improved, as indicated by the negative learning slope, even when starting from a low base. By contrast, the worst performers started out slowly and showed little improvement. They were characterized by high asymptotes and high data variability. The remaining trainees performed irregularly across trials, sometimes improving and sometimes worsening from trial to trial, indicated by rises and falls in the learning slope and the wide spread of data. Not all learners improved over the course of the teaching, and learning failed to stabilize during the asymptote (last 5 trials), even when repetitively performing tasks at the same site on the same cadaver.

Unlike Dreyfus and Dreyfus [29], we identified 4 rather than 5 disparate groups for both scanning and needling phases using cluster analysis techniques. No previous anesthesia studies have attempted to measure and categorize skills using the Dreyfus criteria. Therefore, our results provide a unique insight into the range of interscalene block skills from novice to expert and broadly reflect the descriptors defined by Dreyfus and Dreyfus [29].

Nevertheless, it must be borne in mind that we only defined the range of skills for a single interscalene block. We hypothesize that when faced with a new cadaver, new patient, or new block, even more learning will be required, and fewer participants will be likely to plateau or match the performance of experts.

We recommend that skills training be built in a series of isolated steps so that performance can be measured while new skills are being acquired. We do not take the view that one course of mastery learning is sufficient, but that ongoing training and assessment are required to ascend the skills ladder proposed by Dreyfus and Dreyfus [29] and reduce the effects of skill drain. In future work, we intend to measure the rate of skill loss after training as a means of timing the need for retraining on the simulator.

### Metrics

Third, we demonstrated a strong construct validity with respect to attentional focus. Most studies have demonstrated construct validity by comparing novices or even nonanesthesiologists to experts. In contrast, using a heterogeneous group of anesthesiologists, our study showed an improvement in attentional focus over several groups in the following order: experts>fellow>higher trainees>intermediate trainees>novice trainees (Figure 3). Experts focused intently on the monitor, had fewer fixations, shorter overall dwell time (less time spent attending to the screen or tool area), and fewer switches in attention between the target and tools compared with novices. This may indicate novice difficulty in handling tools (probe or needle) or greater cognitive processing. Some trainees were recruited after finishing the night on call and may have been tired. We admit that the failure to standardize trainee wakefulness left us with some background statistical *noise*. Nevertheless, we feel that this study at least represented everyday practice, and we were readily able to expose the variability and discriminatory properties of eye tracking metrics.

### Simulator

Fourth, our study was made possible using our durable cadaver simulator. Unlike fresh frozen cadavers, the soft embalmed cadaver tolerated 934 injections over 2 weeks [16]. The fluid quickly drained away from the interscalene groove and provided good conditions for repetitive practice. Needle tracks were not seen, and external pressure marks resolved because the cadaver retained its elasticity. Images demonstrating this phenomenon are available in our previous publication [17]. Cadaver hire is not inexpensive, costing £250 (US $300) per half-day of training, although it is possible for 2 training groups to be accommodated at either end of the cadaver. Our group has already successfully executed 4 courses to enhance proficiency by using mastery methods.

We chose to restrict the number of cadavers used. We expected large intraparticipant variance as performance improved with repetition (learning curve), and we foresaw large interparticipant variance because we were examining the widest possible range of skills between anesthesiologists. Therefore, the use of several cadavers would have introduced even more variance and necessitated a large study.

However, we do appreciate that the restriction of cadavers does not reflect the variation seen in clinical practice, and we would urge some caution when extrapolating data. We intend to conduct an RCT that also exposes participants to multiple cadavers and study transference by allocating them to multiple nerve blocks.

Of interest to teachers is how many blocks does it take to attain the expertise of experts? To calculate this, one may assume a relationship between time (T) and the number of blocks (N) as a negative power learning curve in the form: T=b* N^-^a. *or^a^ N^-^a ^= b^/^T.^*

From our data, assuming *b*=60 and *a*=0.005 derived from fitting curves to our data, expertise equivalent to our fellow is gained after 189 blocks and needs 284 or 550 blocks to resemble the performance of experts 2 and 1, respectively. Increasing *b* to 70 or 80 increases the requirement to 351 and 377, respectively, to match consultant 2.

### Comparison With Prior Work

Our findings are consistent with the expert behavior observed in laparoscopy, radiology, and chess playing [9], and this is thought to be underpinned by the gradual build-up of memories of visual structures. We hypothesized that dedicated practice in a cadaver-based mastery learning environment provides an opportunity to repeatedly encode these visual memories. The best novices were more experienced and approached the performance of the regional fellow but not expert consultants. We suggest that experienced novices probably developed transferable skills from the general experience of ultrasonography, for example, during central line insertion.

Trainees characteristically tend to be *overloaded* with information psychomotor performance, spatial judgments, monitoring data, instruction, and intraoperative events [30,31]. Limited cognitive resources are available to enable decisive and correct decision-making. Thus, the automation of technical skills through mastery learning and feedback enables trainees to cope with the challenges of regional anesthesia by minimizing cognitive overload.

Two studies used eye tracking in UGRA and demonstrated, as in our study, greater attentional focus on targets during simulated tasks with experts than trainees [4,32]. Surgical disciplines have made progress in the application of attentional theory and practice to medical training. In laparoscopy training, experts achieved faster task completion in simulator training, greater attentional focus to the target, and fewer switches between the target and tools [33,34]. What is missing from the literature is the study of incremental steps toward competency and intermediate expertise.

### Future Directions

Our results suggest that eye tracking can be regarded as a means of formative feedback (via visual feedback on gaze behavior) and assessment within the context of a mastery learning program. It provides a deeper understanding of why trainees learn at different rates based on their attentional patterns and allows reflection on performance. We suggest that an objective assessment of performance using eye movements can complement traditional methods by reducing assessment variability between trainers. Ultimately, this method could be adapted from face-to-face learning to remote web-based education.

Real-time eye tracking metrics are not yet available because the data must be analyzed by a statistician. With this in mind, our team is developing algorithms that would allow the translation of measurements to clinical environments and correlate eye tracking with motion analysis.

### Conclusions

Our collaborative, translational approach to measuring technical skills performance fits well with recommendations within the recent Topol Report on Digital Medicine [35]. We have shown substantial improvements in skill acquisition and present data that demonstrate how technology can be used to quantify complex human performance.

## Abbreviations

CAHID: Centre for Anatomy and Human Identification
IPIP: International Personality Item Pool
LOESS: Locally Weighted Scatterplot Smoother
ST: specialist trainee
UGRA: ultrasound-guided regional anesthesia
